# The emerging role of miRNAs in the pathogenesis of COVID-19: Protective effects of nutraceutical polyphenolic compounds against SARS-CoV-2 infection

**DOI:** 10.7150/ijms.76168

**Published:** 2022-07-18

**Authors:** Chih-Yun Yang, Yu-Hsuan Chen, Pei-Jung Liu, Wan-Chung Hu, Kuo-Cheng Lu, Kuo-Wang Tsai

**Affiliations:** 1 Division of Chest Medicine, Kaohsiung Municipal Min-Sheng Hospital, Kaohsiung, Taiwan, ROC; 2 Division of Chest Medicine, Department of Internal Medicine, CHENG HSIN General Hospital, Taipei, Taiwan, ROC; 3 Division of Chest Medicine, Department of Internal Medicine, Kaohsiung Veterans General Hospital, Kaohsiung, Taiwan, ROC; 4 Department of Clinical Pathology and Medical Research, Taipei Tzu Chi Hospital, Buddhist Tzu Chi Medical Foundation, New Taipei City, Taiwan, ROC; 5 Division of Nephrology, Department of Medicine, Taipei Tzu Chi Hospital, Buddhist Tzu Chi Medical Foundation, New Taipei City, Taiwan, ROC; 6 Division of Nephrology, Department of Medicine, Fu-Jen Catholic University Hospital, School of Medicine, Fu-Jen Catholic University, New Taipei City, Taiwan, ROC; 7 Department of Research, Taipei Tzu Chi Hospital, Buddhist Tzu Chi Medical Foundation, New Taipei City, Taiwan, ROC

## Abstract

Severe acute respiratory syndrome coronavirus 2 (SARS-CoV-2) infection can cause immunosuppression and cytokine storm, leading to lung damage and death. The clinical efficacy of anti-SARS-CoV-2 drugs in preventing viral entry into host cells and suppressing viral replication remains inadequate. MicroRNAs (miRNAs) are crucial to the immune response to and pathogenesis of coronaviruses, such as SARS-CoV-2. However, the specific roles of miRNAs in the life cycle of SARS-CoV-2 remain unclear. miRNAs can participate in SARS-CoV-2 infection and pathogenesis through at least four possible mechanisms: 1. host cell miRNA expression interfering with SARS-CoV-2 cell entry, 2. SARS-CoV-2-derived RNA transcripts acting as competitive endogenous RNAs (ceRNAs) that may attenuate host cell miRNA expression, 3. host cell miRNA expression modulating SARS-CoV-2 replication, and 4. SARS-CoV-2-encoded miRNAs silencing the expression of host protein-coding genes. SARS-CoV-2-related miRNAs may be used as diagnostic or prognostic biomarkers for predicting outcomes among patients with SARS-CoV-2 infection. Furthermore, accumulating evidence suggests that dietary polyphenolic compounds may protect against SARS-CoV-2 infection by modulating host cell miRNA expression. These findings have major implications for the future diagnosis and treatment of COVID-19.

## Introduction

Coronavirus disease 2019 (COVID-19) has emerged as a new epidemic disease caused by severe acute respiratory syndrome coronavirus 2 (SARS-CoV-2) infection 1. COVID-19 has spread rapidly and placed tremendous strain on global health-care systems. SARS-CoV-2 targets angiotensin-converting enzyme 2 (ACE2) receptors in human lung and gastrointestinal tissues. The receptor-binding domain (RBD) of the SARS-CoV-2 spike (S) protein binds to ACE2 on the plasma membranes of infected cells, initiating receptor-mediated endocytosis. SARS-CoV-2 variants, such as the Delta and Omicron variants, have distinct viral S (S1 or S2) proteins and RBDs, which may make these variants more infectious by enhancing their affinity for ACE2 2, 3. The high infectivity of these new variants has led to the acceleration of the spread of COVID-19 worldwide. Most patients with COVID-19 exhibit some typical clinical symptoms, such as fever, fatigue, cough, expectoration, sputum production, and shortness of breath. Children with COVID-19 tend to present with similar symptoms, which are usually milder than those experienced by adult patients 4, 5. Older adult patients with COVID-19 have a higher frequency of severe illness and COVID-19-related death than do younger patients 5. These findings suggest that comorbidities and age may be crucial factors in determining the severity of COVID-19.

Several strategies for blocking the interaction between SARS-CoV-2 and ACE2 receptors, thus preventing the spread of infection, have been developed. Soluble RBD mimetics or antibodies against ACE2 receptors bind to ACE2 receptors and prevent the virus from binding to and entering host cells. A similar effect can be achieved by directly targeting and binding to the RBDs of coronavirus S proteins by using the extracellular domain of ACE2 as bait 6. SARS-CoV-2 infection may induce the expression of several host cell genes, which may produce antiviral or proviral effects and even help the virus evade the immune response 7. Therefore, several anti-SARS-CoV-2 drugs, including such as favipiravir, remdesivir, lopinavir, ritonavir, chloroquine, ribavirin, and umifenovir, have been developed to prevent viral entry into host cells and suppress various steps of viral replication 8. However, the clinical efficacy of these drugs for COVID-19 remains limited.

Numerous treatments and vaccines for COVID-19 have already been developed; however, the detailed molecular pathogenic mechanisms must be further elucidated to facilitate the development of new, more effective therapeutics against SARS-CoV-2 variants. microRNAs (miRNAs) are small noncoding RNA molecules that regulate the expression of genes posttranscriptionally. Recent studies have confirmed that host and viral miRNAs are necessary for successful SARS-CoV-2 infection. Several miRNAs can inhibit the expression of proteins involved in the SARS-CoV-2 life cycle, such as ACE2, TMPRSS2, S proteins, and Nsp12. Therefore, miRNAs acting on the viral entry pathway may serve as potential therapeutic tools against COVID-19 9. Naturally occurring nutraceutical polyphenolic compounds such as resveratrol have antioxidant, antitumor, antiviral, and free radical-scavenging properties; therefore, they may be used as adjunctive therapy for COVID-19 10. Preclinical studies have revealed that resveratrol has promising effects for the treatment of COVID-19 11. Polyphenolic compounds also interact with cellular signaling pathways, regulate gene and miRNA expression, affect the activity of transcription factors 12. The use of harmless nutraceuticals to inhibit viral entry into cells and viral replication by modulating host cell entry-related miRNAs is a feasible approach to addressing the emergence of new SARS-CoV-2 variants. In this review, we present recent findings on the role of miRNAs in SARS-CoV-2 cell entry. We also describe various polyphenols that are widely used as nutraceuticals and explore the epigenetic ability of these compounds to modulate miRNA expression and to potentially prevent SARS-CoV-2 cell entry and viral replication 12.

## MicroRNA biogenesis and mechanisms

MicroRNAs (miRNAs) are small RNAs with critical functions in several physiological processes 13. Mature miRNAs are produced from primary transcripts (pri-miRNAs), which are processed by Drosha proteins into precursor miRNAs (pre-miRNAs), each of which consists of 5p and 3p arms and a terminal loop. The pre-miRNAs are transported to the cytoplasm by exportin 5 and are divided by Dicer to release the terminal loop and 5p/3p duplex. Finally, according to the hydrogen bond theory, the 5p or 3p arm is selectively loaded onto the RNA-induced silencing complex 14-16. However, studies have reported different arm selection preferences of miRNA (for the 5p or 3p arms) in different tissues, developmental stages, and species and during cancer progression 17-25. Researchers have hypothesized that these arm selection preferences are regulated by target-mediated miRNA protection 26, 27. MiRNA performs its biological functions by targeting the 3′-untranslated region (3′-UTR) of protein-coding genes to degrade mRNA or inhibit protein translation. Therefore, an single pre-miRNA structure can generate two mature miRNAs, miR-#-5p and -3p, which might have distinct functions in distinct cell types or under distinct physiological conditions 28. miRNAs play key roles in antiviral responses and viral pathogenesis in hosts infected with herpesviruses, polyomaviruses, retroviruses, pestiviruses, and hepatitis viruses as well as coronaviruses 29. The induction of miRNA expression by viral infection boosts the immune response by altering the gene expression profiles of host cells. The miRNAs induced by these viruses can be used to identify targets involved in the viral life cycle. Therefore, several studies have developed and evaluated the use of virus-induced miRNAs in antiviral therapies, including treatments for human immunodeficiency virus 1, herpes simplex virus, dengue fever, influenza, and hepatitis C 30.

## Life Cycle of SARS-CoV-2

SARS-CoV-2 is a single-stranded positive RNA virus with a large genome (approximately 29.9 kb in length), which comprises a variable number of open reading frames encoding 16 nonstructural proteins (NSPs) as well as structural proteins (including spike [S], envelope [E], membrane [M], and nucleocapsid [N] proteins) and six accessory proteins (3a, 6, 7a, 7b, 8, and 10) 22. MiRNAs derived from host cells may have a positive or negative effect on the viral life cycle and viral replication by directly binding to genomic or transcriptional SARS-CoV-2 RNA. These SARS-CoV-2-regulated host miRNAs can affect the host's immune system by indirectly regulating the expression of key genes in the message transmission pathway 31.

**Figure 1:**
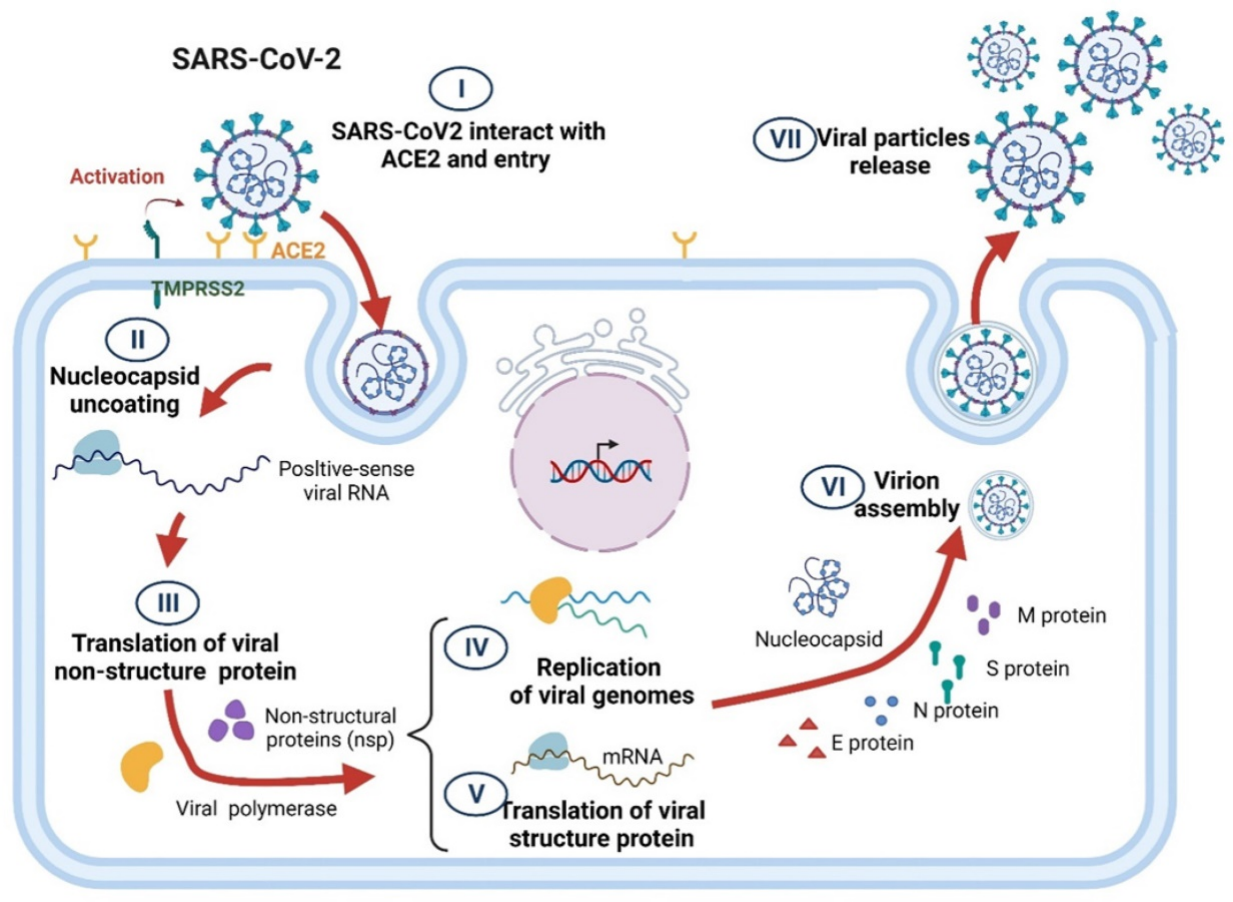
Schematic of SARS-CoV-2 life cycle.

The life cycle of SARS-CoV-2 comprises several steps, including virus particle attachment, entry into host cells, genome replication, essential protein synthesis, viral component assembly, and release. In brief, transmembrane serine protease 2 (TMPRSS2) causes SARS-CoV-2 S proteins to interact with the angiotensin-converting enzyme 2 (ACE2) surface receptors of a host cell, thereby enabling the viruses to enter the host cell through endocytosis 32. After proteolysis, the viral RNA is released, which initiates the viral genome replication and the translation of essential viral proteins using the host cell's replication and protein translation machinery. Numerous structural proteins are rapidly produced, and viral components, including the viral RNA genome and structural proteins, are packaged into SARS-CoV-2 particles and released through exocytosis to infect surrounding cells (Figure 1) 33, 34.

## MicoRNA participate in SARS-CoV-2 pathogenesis

In human cells, miRNAs can also play a role in preventing viral infection by blocking target pathways required for viral penetration, replication, and spread, including the p38 MAPK, PI3K/Akt, FAK, IFN-gamma, TGF-beta, interleukin, IGF1, and TRAIL signaling pathways 7, 35-37. Khan et al. identified three miRNAs (hsa-miR-17-5p, hsa-miR-20b-5p, and hsa-miR-323a-5p) that exhibit antiviral effects against SARS-CoV-2 during host infection 7. Some miRNAs, including hsa-miR-8066, hsa-miR-5197-3p, and hsa-miR-3934-3p, modulate the SARS-CoV-2 life cycle by influencing biosynthesis 38. Sato et al. reported that hsa-miR-15b-5p interacts with RNA polymerase, which might affect SARS-CoV-2 replication 39. A comprehensive analysis of the cell-free RNA profiles of the plasma of patients with COVID-19 and healthy controls revealed that the expression of hsa-let-7 family members, hsa-miR-23a-3p, hsa-miR-23b-3p, hsa-miR-451a, and hsa-miR-316 in patients with COVID-19 was significantly lower than that in healthy individuals. The downregulation of these miRNAs in patients with COVID-19 leads to IL-6/IL-6R hyperactivation by directly targeting the 3'UTR of IL-6/IL-6R, thereby enhancing the cytokine storm induced by SARS-CoV-2 infection 40.

**Figure 2:**
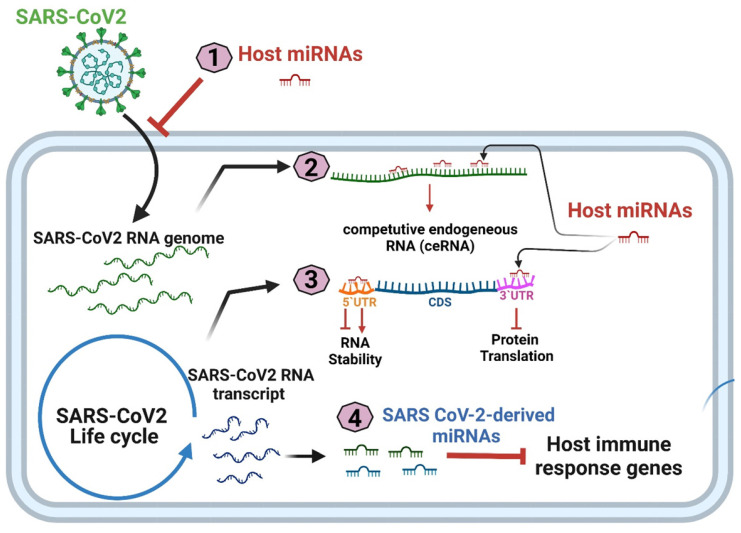
Four putative mechanisms of miRNA participation in SARS-CoV-2 pathogenesis.

Meidert et al. reported that the differential expression of hsa-miR-3168, hsa-miR-146a-5p, and hsa-let-7e-5p in patients with COVID-19 can affect the expression of inflammation-related genes, including IL-6, IL-8, and Toll-like receptor 4 (TLR4)^41^. In addition to influencing inflammation-related pathways, host miRNAs may negatively regulate the viral life cycle by inhibiting viral entry or the translation of the viral genome; alternatively, the binding of miRNAs to the 5′ or 3′UTR may lead to greater RNA stability and increased viral replication 42. Overall, miRNAs participate in SARS-CoV-2 infection and pathogenesis through four mechanisms: (1) host cell miRNA expression interfering with SARS-CoV-2 cell entry; (2) SARS-CoV-2-derived RNA transcripts acting as competitive endogenous RNAs (ceRNAs) that may attenuate host cell miRNA expression; (3) host cell miRNA expression modulating SARS-CoV-2 replication, and (4) SARS-CoV-2-encoded miRNAs protecting SARS-CoV-2 from degradation and silencing the expression of host protein-coding genes (Figure 2).

### Host cell miRNA expression interferes with SARS-CoV-2 cell entry

**Figure 3:**
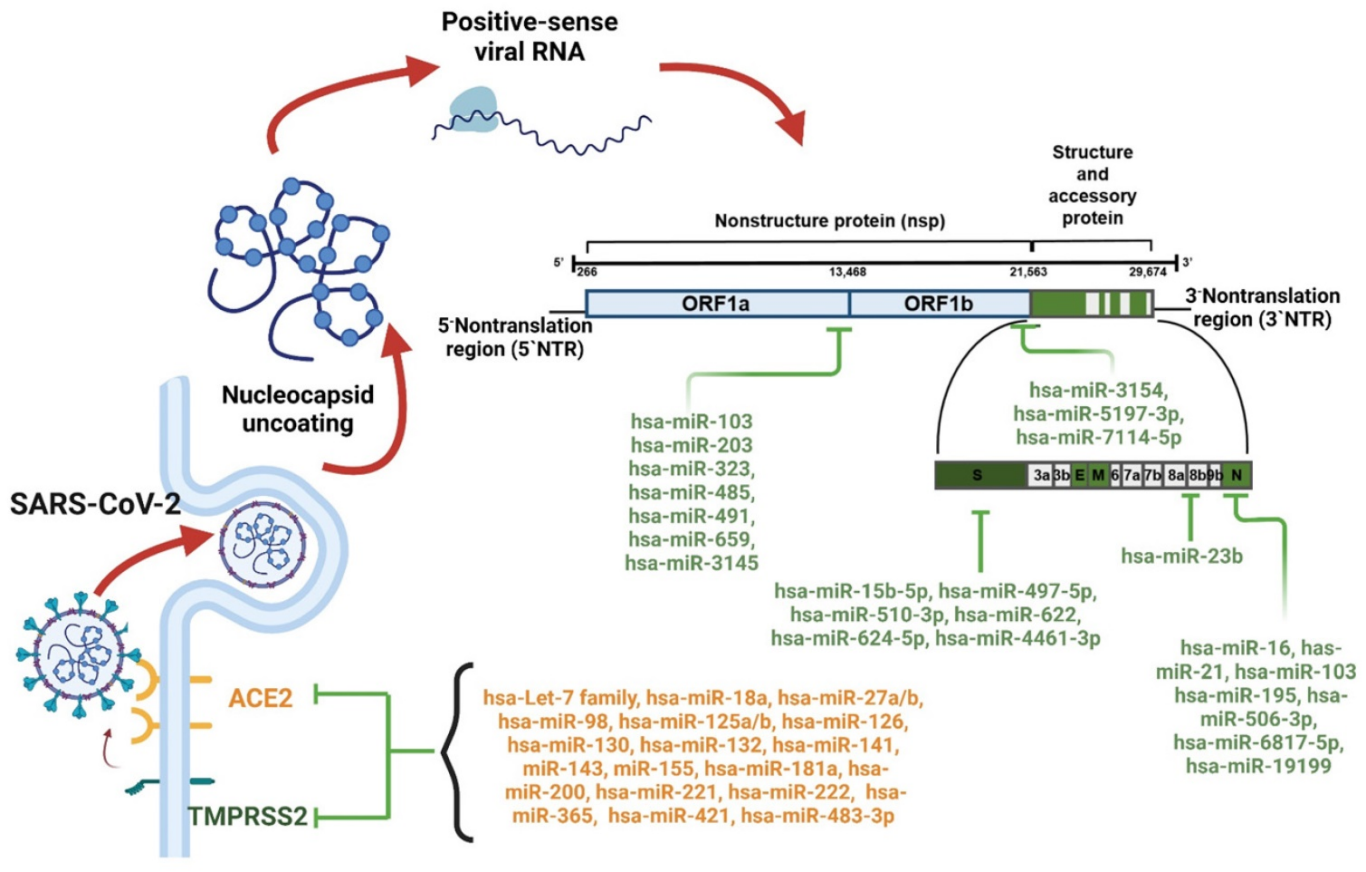
Human miRNAs regulating SARS-CoV-2 infection and replication.

Numerous human miRNAs have binding sites across the 3′UTRs of *ACE2* and *TMPRSS2* and therefore exhibit antiviral effects (Figure 3 and Table 1). Hsa-miR-200c inhibits ACE2 by targeting *ACE2* mRNA in primary cardiomyocytes, which suggests that hsa-miR-200c could reduce the risk of SARS-CoV-2 infection 43. In addition, hsa-let-7e and hsa-miR-98-5p could suppress *TMPRSS2* gene expression by directly targeting the 3'UTR of TMPRSS2 44, 45. Nersisyan et al. reported that hsa-miR-125a, hsa-miR-141, and hsa-miR-200 family members could regulate ACE2 expression by directly targeting the 3'-UTR of ACE2 45. A similar study revealed that the host miRNAs hsa-miR-9-5p and hsa-miR-218-5p could target the 3'-UTR of ACE2 and that hsa-Let-7d-5p, hsa-Let-7e-5p, hsa-miR-494-3p, hsa-miR-382-3p, and hsa-miR-181c-5p could target the 3'-UTR of TMPRSS2 46. Hsa-miR-18 and hsa-miR-125b play central roles in acute renal injury in patients with SARS-CoV-2 infections by directly binding to ACE2. Furthermore, anti-hsa-miR-18 and anti-hsa-miR-125b are effective against SARS-CoV-2 infection 47.

### SARS-CoV-2-derived RNA transcripts act as competitive endogenous RNAs (ceRNAs) that may attenuate host cell miRNA expression

RNA transcripts perform crucial biological functions by interacting with and titrating the expression of endogenous miRNAs 48, 49. Therefore, both the RNA transcripts and genomic RNA of SARS-CoV-2 can act as ceRNA to regulate the activity of endogenous miRNAs. Host-pathogen interactions via host cellular components play a major role in viral infection. Viral miRNAs have been identified as key players in host-virus interactions. In addition to protein-coding mRNAs, noncoding RNAs may be targeted in infected cells, and viruses can exploit the host miRNA network through the ceRNA effect 50. ceRNAs are transcripts that can regulate each other posttranscriptionally by competing for shared miRNAs. Because any transcripts harboring miRNA bonding sites can theoretically function as ceRNAs; they represent a widespread form of posttranscriptional regulation of gene expression in both physiology and pathology 51. Accumulating evidence indicates that ceRNA networks link the functions of protein-coding mRNAs with those of noncoding RNAs such as miRNAs, long noncoding RNAs 52, pseudogenic RNAs, and circular RNAs 51, thereby affecting and regulating the expression of target genes. Because host miRNAs can bind to the coding DNA sequence (CDS) regions of viral RNAs, even without interfering with viral RNA function, overconsumption of host miRNAs (known as the sponge effect) may lead to a reduction in the availability of such miRNAs, thus increasing the severity of COVID-19 infection. Using target prediction tools, numerous studies have identified putative antiviral host miRNAs targeting virus genes. Haddad et al. identified 10 miRNAs (hsa-miR-4288, hsa-miR-195-5p, hsa-miR-16-5p, hsa-miR-15b-5p, he-miR-15a-5p, hsa-miR-6838-5p, hsa-miR-497-5p, hsa-miR-424-5p, hsa-miR-3133, and hsa-miR-21-3p) that can bind to the single-stranded RNA of the full-length SARS-CoV-2 genome. Among these miRNAs, hsa-miR-510-3p, hsa-miR-624-5p, and hsa-miR-497-5p exhibit high potential to target the mRNA encoding the S glycoprotein of SARS-CoV-2 53. Hsa-miR-15b-5p, hsa-miR-151a-5p, hsa-miR-196a-5p, hsa-miR-380-5p, hsa-miR-449a, hsa-miR-565, hsa-miR-622, and hsa-miR-761 target the S protein gene 54. Four human miRNAs (hsa-miR-4464, hsa-miR1234-3p, hsa-miR-7107-5p, and hsa-885-5p) exhibit perfect complementarity with the RBD of the S gene of SARS-CoV-2 55. Nersisyan et al. performed computational prediction to identify critical miRNAs that interact with human coronaviruses and identified six miRNAs (hsa-miR-21-3p, hsa-miR-195-5p, hsa-miR-16-5p, hsa-miR-3065-5p, hsa-miR-424-5p, and hsa-miR-421) that exhibited high binding probability across all the analyzed viruses 56. Another study, which used 67 different SARS-CoV-2 genomes to identify conserved miRNA binding sites, revealed that 10 miRNAs (hsa-miR-103a-1-5p, hsa-miR-6818-5p, hsa-miR-624-5p, hsa-miR-378c, hsa-miR-3202, hsa-miR-5591-5p, hsa-miR-8082, hsa-miR-939-5p, hsa-miR-549a-3p, and hsa-miR-6515-5p) can bind to the ORF1a/b region of the SARS-CoV-2 genome 7.

Using the bioinformatics approach, Chow et al. identified 128 human miRNAs with the potential to bind to the genomic RNA of SARS-CoV-2 38. Among them, the expression levels of six miRNAs differed significantly between lung cells infected with SARS-CoV-2 and healthy lung cells: the expression levels of hsa-Let-7a-3p, miR-16-2-3p, hsa-miR-135-5p, and miR-1275 were significantly downregulated in the SARS-CoV-2-infected lung cells, whereas those of hsa-miR-139-5p and hsa-miR-155-3p were significantly upregulated 38. Using various target prediction tools, Fulzele et al. identified 873 human miRNAs targeting the SARS-CoV-2 genome 57. Pathway enrichment analysis revealed that these miRNAs are involved in various age-related signaling pathways. The authors' findings indicate that older adult patients with SARS-CoV-2 infections tend to have greater disease severity and higher mortality rates than do younger patients because of low miRNA expression, which is directly suppressed through sponging. Another team identified 479 human miRNAs that could bind to the SARS-CoV-2 genome. Among them, 369 targeted an ORF1a/b sequence 58. Taken together, the results of these three studies indicate that 11 human miRNAs (hsa-miR-15b-3p, hsa-miR-19b-1-5p, hsa-miR-19b-2-5p, hsa-miR-125a-3p, hsa-miR-141-3p, hsa-miR-153-5p, hsa-miR-196a-5p, hsa-miR-1202, hsa-miR-1301-3p, hsa-miR-4758-5p, and hsa-miR-5047) may target sequences of SARS-CoV-2.

**Figure 4:**
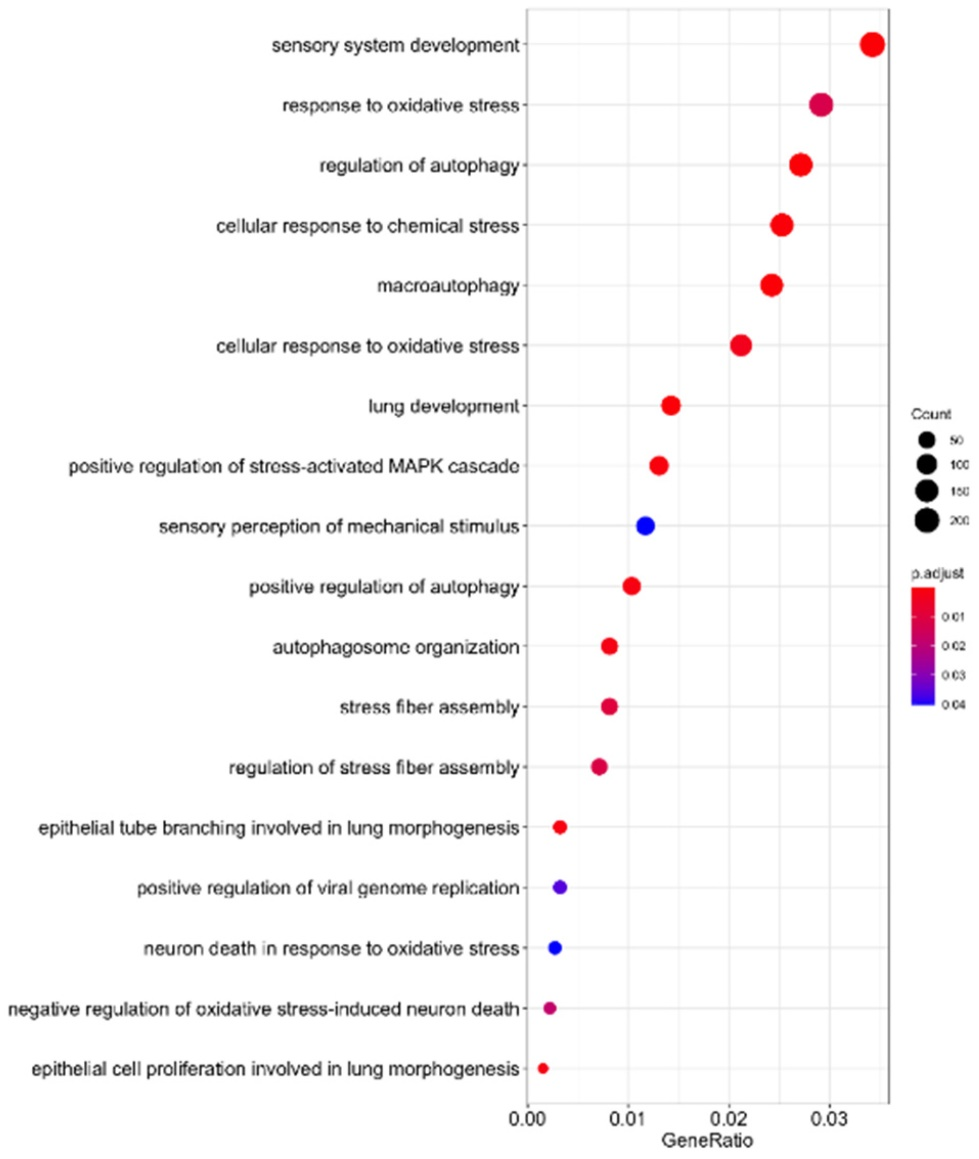
SARS-CoV-2 RNA sponging of miRNAs involved in human signaling transduction pathways.

As indicated in Table 1, 41 microRNAs have been determined to target sequences of SARS-CoV-2, suggesting that these human miRNAs might be sponged during SARS-CoV-2 replication. To elucidate the functions of these sponged host miRNAs, we identified their putative target genes and conducted pathway enrichment analysis. As indicated in Figure 4, these miRNAs are involved in sensory system development, oxidative responses, autophagy, lung development, stress-activated MAPK signaling, and oxidative stress-induced neuron death. A previous study reported that patients with SARS-CoV-2 infection have excessive reactive oxygen species (ROS) levels, which facilitates the cascade of biological events that drive pathological host responses 59. High ROS levels induce tissue damage, thrombosis, and red blood cell dysfunction, which contribute to the severity of COVID-19. Macroautophagy and autophagy are crucial to viral replication and maturation 60. According to RNA sequencing data of SARS-CoV-2-infected cells, MAPK signaling is associated with lung fibrosis, a lethal complication of COVID-19 61. These findings demonstrate that SARS-CoV-2 RNA sponging host endogenous miRNAs is crucial to COVID-19 progression.

### (3) Host cell miRNA expression modulates SARS-CoV-2 replication

In addition to affecting viral entry into host cells, many host miRNAs also target and suppress the replication, translation, and protein synthesis-related gene expression of SARS-CoV-2. hsa-miRNA can bind to the SARS-CoV-2 genome. Host miRNAs can facilitate viral replication by targeting the viral 5′ noncoding region or by controlling (repressing) the translation of viral mRNA into proteins by targeting the viral 3′ noncoding region, thus enabling the virus to evade the host immune system 62, 63. Briefly, miRNA binding to 5′ UTR leads to RNA stability and increased viral replication. miRNA binding to 3′ UTR can lead to the inhibition of viral translation or increased RNA stability and viral translation. The SARS-CoV-2 gene ORF6 and CDSs NSP13, NSP14, and NSP15 can powerfully suppress both primary interferon production and interferon signaling. Among the 27 known viral proteins of SARS-CoV-2, ORF6 most strongly suppresses both primary interferon production and interferon signaling 64. SARS-CoV-2 ORF6 can also act as a virulence factor by regulating nucleocytoplasmic trafficking to accelerate viral replication, resulting in rapid disease progression 65. Certain human miRNAs (including miRNA-323 and miRNA-485) target ORF1a/b, which encodes enzymes necessary for the replication and translation of SARS-CoV-2 66. Many human miRNAs modulate the life cycle of SARS-CoV-2 by directly targeting the RNA encoding NSPs or structural proteins (Figure 3 and Table 1). Chen et al. identified two miRNAs (hsa-miR-1307-3p and hsa-miR-3613-5p) that could prevent viral replication by targeting the 3'-UTRs of replication-related SARS-CoV-2 RNA 67. Hsa-miR-203b-3p can suppress viral replication by targeting the sequence of ORF1ab and ORF3a 58. The high abundance of hsa-miR-497-5p, hsa-miR-21-3p, and hsa-miR-195-5p were predicted targeted at SARS-CoV-2 genome, that degrade the RNA of coding-region genes of SARS-CoV-2, thereby suppressing SARS-CoV-2 replication 53. By acting as host miRNA sponges, noncoding SARS-CoV-2 RNAs may dysregulate and suppress the expression of human miRNAs, including hsa-miR-10a-5p, hsa-miR-99b-5p, hsa-miR-376a-3p, and hsa-miR-548a-5p, which are involved in immune responses 68. Bartoszewski *et al.* reported that hsa-miR-34a/c-5p, hsa-miR-92a-5p, hsa-miR-138-5p, hsa-miR-449c-5p, hsa-miR-766-5p, hsa-miR-3940-5p, and hsa-miR-6741-5p can bind to multiple sites on noncoding SARS-CoV-2 RNA, which can affect the host immune response 68. Circulating hsa-miR-150-5p plays a crucial role in inhibiting SARS-CoV-2 infection by directly interacting with the nsp10 region of SARS-CoV-2 RNA 69.

### (4) SARS-CoV-2-encoded miRNAs protect SARS-CoV-2 from degradation and silencing the expression of host protein-coding genes

Studies have identified multiple DNA and RNA viruses that produce pre-miRNA sequences that may undergo further maturation induced by the human RNA-induced silencing complex 70, 71. Viral miRNAs have been identified in many human viruses, including influenza 72, EV71 73, hepatitis A 74, and SARS-CoV 75, 76. The structure and function of these viral miRNAs are highly similar to those of human miRNA. It is well known that the transcription and translation mechanisms of the host cell are necessary for viral replication, translation, and protein synthesis 77, 78. Viral miRNAs protect SARS-CoV-2 mRNA from turnover and degradation in human cells by inhibiting certain host mRNA deadenylases. Viral miRNAs also target transcriptional regulators to prevent RNA polymerase II from attaching to the promoters of host genes 58. Morales et al. used deep RNA sequencing to profile the expression of small RNAs in the lungs of SARS-CoV-2 infected mice. They identified three SARS-CoV-derived miRNAs, which were derived from the nsp3 (svRNA-nsp3.1 and -nsp3.2) and N (svRNA-N) genomic regions of SARS-CoV 75. Using antagomir to block SARS-CoV-derived miRNAs could significantly reduce in vivo lung pathology and proinflammatory cytokine expression, which indicates that virus-derived miRNA may be a therapeutic target in patients with SARS-CoV infections. Arisan *et al.* identified seven key-microRNAs (miR-8066, miR-5197, miR-3611, miR-3934-3p, miR-1307-3p, miR-3691-3p, and miR-1468-5p) with sequences similar to those of human miRNA and SARS-CoV-2 79. These SARS-CoV-2-derived miRNAs could completely complement the target RNA transcripts of SARS-CoV-2 to prevent viral replication and protein translation. Recently, Fu et al. identified a SARS-CoV-2-derived miRNA, miR-nsp3-3p, that was encoded at nucleotides 3874-2894 on the 3'-UTRs of nsp3 genes 80. Furthermore, the expression levels of miR-nsp3-3p can effectively predict critical illness risk and outcomes (as related to disease progression and recovery) in patients with COVID-19. Aydemir et al. used Kyoto Encyclopedia of Genes and Genomes (KEGG) pathway analysis and gene regulatory networks to identify 40 SARS-CoV-2-encoded miRNAs and their regulatory targets, some of which play key roles in the NFκB, JAK/STAT, and TGFβ signaling pathways and cellular epigenetic regulation pathways 81. One study identified 27 SARS-CoV-2-encoded miRNAs that bind to regions of the SARS-CoV-2 genome. Most of the target sites of these miRNAs are located on the ORF1ab gene, and some sites are located in the 5'-UTR of the viral genome and the S gene. Virus-encoded miRNAs that bind to genomic regions can regulate viral replication and host entry 66. Conversely, SARS-CoV-2-encoded miRNAs can also act on the host genome. miR-147-3p, encoded by SARS-CoV-2, enhances the expression of *TMPRSS2* in the gut and increases the virus's ability to spread 55, and miR-5197, miR-8066, and miR-3934-3p are involved in the N-linked and O-linked glycosylation of subunit S1 and S2 proteins, which can increase the pathogenicity of SARS-CoV-2 79. Although most miRNAs have conserved sequences, some mutations were detected in miR-1307-3p and miR-1468-5p of SARS-CoV-2 strains, which may be involved in their pathogenicity 82. However, targeting miRNAs that are highly conserved in all SARS-CoV-2 strains appears to be an effective preventive approach and a promising therapeutic strategy.

## Circulating miRNAs act as biomarkers for COVID-19

Several circulating miRNAs have been detected in sera, plasma, urine, tears, amniotic fluid, and gastric juice 83-85. These circulating miRNAs are highly stable in body fluids and might originate from different cell types under different physiological conditions 84, 86. Therefore, miRNAs might be useful noninvasive biomarkers for the diagnosis and prognosis of patients with COVID-19 (Table 2).

## Diagnostic biomarkers

Early diagnosis of COVID-19 and immediate isolation of patients with COVID-19 are effective approaches to preventing the spread of SARS-CoV-2. In addition, early detection of disease deterioration, timely intervention, respiratory support, and nutritional support can effectively reduce the risk of patient mortality. Therefore, identifying potential risk factors for and predicting the course of COVID-19 are crucial for health-care professionals. In general, the nasopharyngeal swab polymerase chain reaction test is the most common tool for COVID-19 diagnosis. However, because active SARS-CoV-2 viruses may be present in the specimens collected from the nasopharyngeal cavity, the collector and examiner might be infected during the testing process. The detection of circulating miRNAs in blood samples would be a safer alternative for COVID-19 diagnosis.

Hsa-miR-10b expression is downregulated in the peripheral blood of patients with COVID-19. Furthermore, hsa-miR-10b expression is significantly and negatively correlated with serum IL-2 and IL-8 in the blood of patients with COVID-19, which suggests that hsa-miR-10b may contribute to cytokine storm 87. In a study comparing the differential miRNA serum profiles patients with COVID-19 and community-acquired pneumonia (CAP), the researched identified four miRNAs (hsa-miR-25-3p, hsa-miR-30a-5p, hsa-miR-106b-5p, and hsa-miR-221-3p) for inclusion in a panel that could be used to significantly discriminate between patients with COVID-19 and CAP 88. Another study demonstrated that the expression of hsa-miR-21, hsa-miR-155, hsa-miR-208a, and hsa-miR-499 in the serum or plasma of patients with COVID-19 was significantly higher than that in the serum of plasma of healthy individuals 89. Receiver operating characteristic (ROC) analysis revealed that hsa-miR-155, hsa-miR-208a, and hsa-miR-499 serum levels could be used to distinguish between COVID-19 and influenza-induced acute respiratory distress syndrome 90. Donyavi et al. conducted an additional ROC analysis and determined that hsa-miR-29a-3p, hsa-miR-146a, and hsa-miR-155-5p expression levels could serve as biomarkers for COVID-19 diagnosis with high specificity and sensitivity 91. McDonald et al. observed that hsa-miR-2392 expression was significantly upregulated in the serum and urine of patients with COVID-19 92. By comprehensively analyzing miRNA profiles, Akula et al. reported that hsa-miR-150-5p, hsa-miR-375, hsa-miR-122-5p, and hsa-miR-494-3p expression levels were significantly upregulated in the plasma of patients with COVID-19, whereas those of hsa-miR-3197, hsa-miR-4690-5p, hsa-miR-1915-3p, and hsa-miR-3652 were significantly downregulated 69. Fayyad-Kazan et al. identified eight miRNAs (hsa-miR-15a-5p, hsa-miR-17-5p, hsa-miR-19a-3p, hsa-miR-19b-3p, hsa-miR-23a-3p, hsa-miR-92a-3p, hsa-miR-142-5p, and hsa-miR-320a) differentially expressed in patients with SARS-CoV-2 and healthy controls. Among them, hsa-miR-19a-3p, hsa-miR-19b-3p, and hsa-miR-92a-3p were combined into a diagnostic panel, which may serve as a diagnostic biomarker with an AUC of 0.917 (*p* =0.0001) 93.

## Prognostic biomarkers

The use of prognostic biomarkers to monitor clinical progress and classify patients can enable health-care providers to provide more precise personalized treatment 94. The reason why some patients with COVID-19 are more likely to experience severe disease symptoms remains unknown. Usually, patients with severe COVID-19 have a high viral load, high oxidative stress, and high levels of inflammatory cytokines 95. COVID-19 can be classified as mild, moderate, severe, or critical. SARS-CoV-2 infection leads to rapid innate immunity activation; therefore, some levels of hematological parameters (white blood cells, lymphopenia, C-reactive protein (CRP), lactate dehydrogenase, and creatine kinase), proinflammatory cytokines (IL-1B, IL-6, IL-8, and G-CSF), and chemokines (MCP1, IP10, and MIP1a) are significantly elevated in the blood of patients with COVID-19 9,10. Elevated levels of these markers are more common among patients with severe COVID-19 than among those with mild COVID-19 and warrant inclusion in risk stratification models.

Using next generation sequencing to profile the small RNA in serum of patients with COVID-19 and heathy controls, researchers identified SARS-CoV-2-derived miR-nsp3-3p as a potential biomarker for the prediction of a patient's risk of severe disease 80. Similar studies showed that hsa-miR-320 family genes, hsa-miR-200c, and hsa-miR-155 are differentially expressed in patients with COVID-19 and are significant correlated with certain clinicopathological characteristics, including CRP, IL-6, and D-dimer levels 89, 96, 97. Hsa-miR-2392 is key to multiple downstream signaling pathways, including those related to inflammation, glycolysis, and hypoxia. High hsa-miR-2392 expression is strongly associated with poor outcomes among patients with COVID-19. Furthermore, anti-hsa-miR-2392 was reported to reduce SARS-CoV-2 viability in both in vivo and in vitro models 92.

Studies have reported that the expression of certain circulating miRNAs in the blood can be used as a biomarker for predicting a patient's response to drug treatment. Sabbatinelli et al. observed low hsa-miR-146a-5p expression in the serum of patients with COVID-19 who did not respond to tocilizumab treatment and determined that low circulating hsa-miR-146a expression in serum was strongly associated with adverse outcomes among patients with COVID-19 98. Another group reported that both hsa-miR-29a-3p and hsa-miR-146a-3p expression could be used to precisely distinguish between acute and post-acute COVID-19 91. A panel of three circulating miRNAs (hsa-miR-148a-3p, hsa-miR-451a and hsa-miR-486-5p) were identified as prognostic biomarkers that could be used to distinguish between patients with COVID-19 admitted to the ICU and those admitted to a clinical ward 99. In addition, the expression of hsa-miR-192-5p and hsa-miR-323a-3p exhibit discretionary potential to predict patient survival 99. Two circulating serum miRNAs, hsa-miR-320b, and hsa-miR-483-5p, were also identified as biomarkers for predicting survival outcomes among patients with COVID-19 100.

### Natural compounds can block SARS-CoV-2 infection by regulating miRNA expression

**Figure 5:**
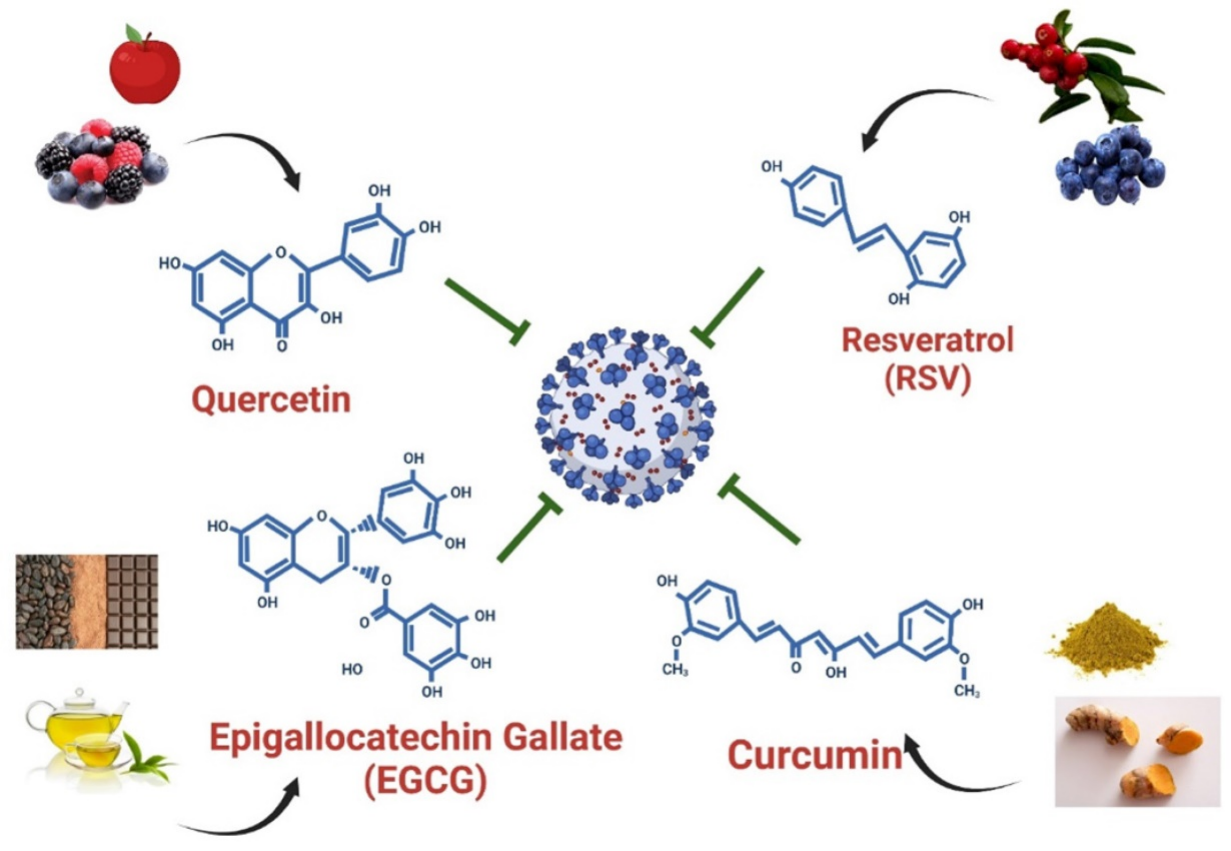
Dietary compounds may protect against SARS-CoV-2 infection. Foods containing quercetin, EGCG, curcumin, and resveratrol and the structures of these polyphenols are illustrated.

Polyphenols are the most abundant dietary antioxidants and are commonly found in fruits, vegetables, chocolate and wine 12, 101, 102. Epidemiological studies have revealed that dietary polyphenol intake can alleviate the symptoms of some chronic diseases, including type 2 diabetes, cardiovascular disease, and COVID-19 103. The progression of COVID-19 is often associated with a high degree of inflammation and overproduction of ROS, resulting in poor outcomes 12, 104. Natural polyphenols are powerful antioxidants and protect against chronic diseases by altering cell signaling pathways and regulating the expression of antioxidant-related genes, indicating that dietary polyphenolic compounds have potential benefits for the prevention of SARS-CoV-2 infection. A limited number of studies have revealed that dietary polyphenolic compounds may act directly against coronaviruses in vitro by modulating expression of host miRNAs 105, 106. In a recent review, authors identified 125 polyphenols-modulating host miRNAs and 644 miRNAs that can interact with SARS-CoV-2 genome by performing literature search 106. Comparison of two groups of miRNA candidates revealed 17 host miRNAs with both capacity to interact with SARS-CoV-2 genome and which expression can be regulate by polyphenols, including hsa-let-7a-3p, has-miR-25-5p, hsa-miR-1246, hsa-miR-125a-5p, hsa-miR-1262, hsa-miR-1290, hsa-miR-148a-5p, hsa-miR-154-3p, hsa-miR-21-3p, hsa-miR-320c, hsa-miR-335-5p, has-miR-34a-5p, hsa-miR-377-5p, hsa-miR-455-3p, has-miR-499a-5p, hsa-miR-544a and hsa-miR-744-3p 106. These polyphenols-induced miRNAs suggested that exerted anti-SARS-CoV-2 capacity by influencing processes of viral replication or entry. In this review, we identified four polyphenols—quercetin, epigallocatechin gallate (ECGC), curcumin, and resveratrol—that have been frequently used as therapeutic nutritional supplements or to prevent SARS-CoV-2 infection (Figure 5). We further searched literatures to demonstrate that four polyphenolic compounds may protect against SARS-CoV-2 infection by modulating host cell miRNA expression.

**Figure 6:**
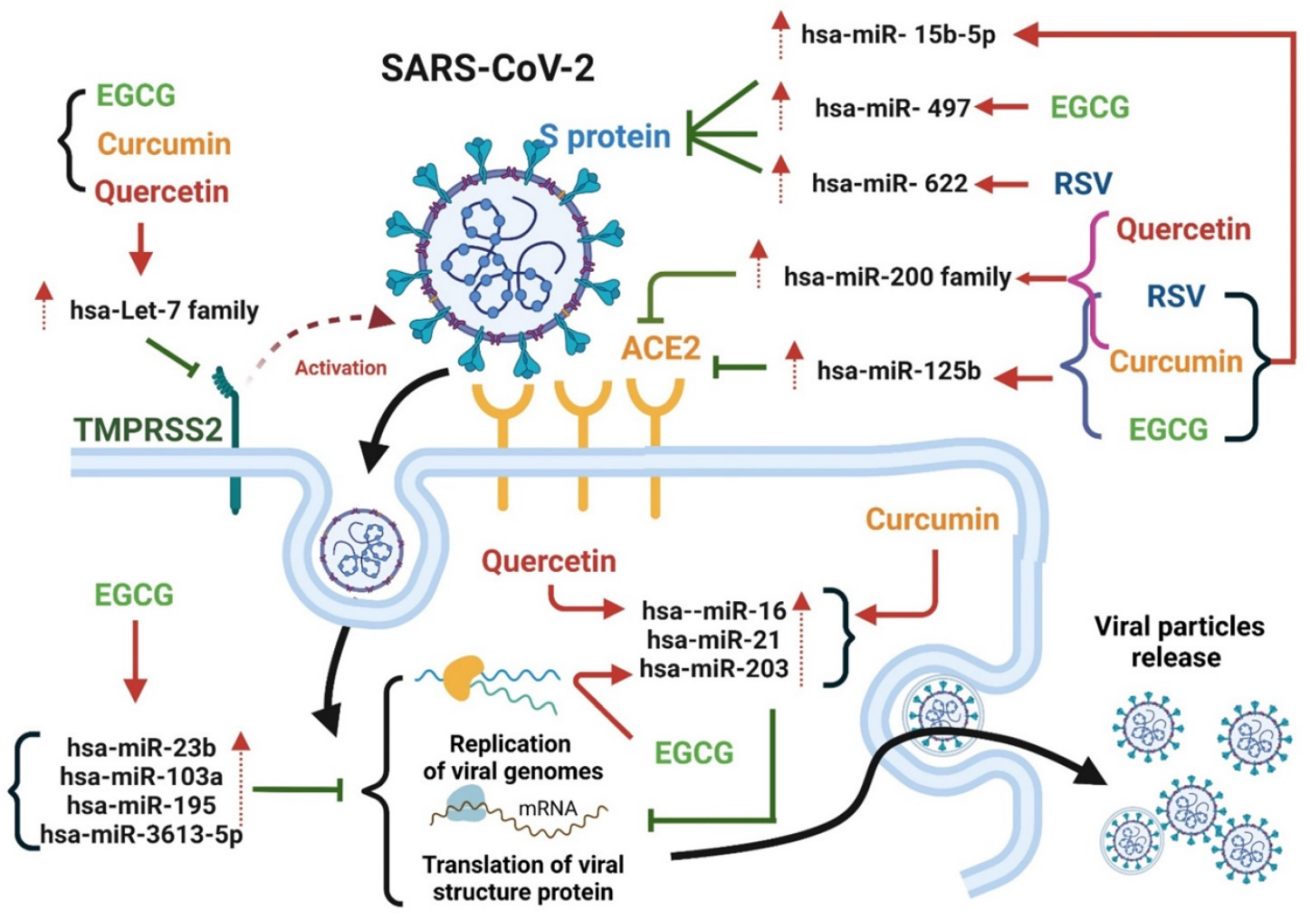
Overview of quercetin-, EGCG-, curcumin-, and resveratrol-mediated inhibition of SARS-CoV-2 entry into host cells and disruption of SARS-CoV-2 life cycle progression through upregulation of miRNA expression.

**Quercetin** is the main representative of the flavonoid subclass of flavanols and is mainly present in fruits such as apples, berries, and grapes (Figure 5). Quercetin is most often present in food as quercetin-3-glucoside (isoquercetin) and is hydrolyzed in the small intestine and rapidly absorbed 107, 108. It exerts antiviral effects by interacting with viral proteins to suppress viral infection, thereby blocking viral replication and entry into host cells 109. Furthermore, quercetin has been determined to be safe and effective in reducing the serum concentrations of ALP, q-CRP, and LDH, and recently, patients with COVID-19 have been treated with daily quercetin supplements in addition to antiviral drugs 110. One study demonstrated that quercetin can effectively inhibit SARS-CoV-2 M^pro^ activity in HuH-7 cells in vitro, with IC_50_ values ranging from 0.125 to 12.9 μM (Table 3) 111. Mangiavacchi et al. reported that two quercetin compounds, quercetin (1) and quercetin (2d), could block SARS-CoV-2 replication in infected cells at nontoxic concentrations, with IC_50_ values of 192 and 8 µM, respectively (Table 3) 112. Accumulating studies have revealed that quercetin may act as a therapeutic agent to suppress human cancer cell growth by regulating miRNA expression 113-115. Following treatment with quercetin, the expression of let-7 family miRNAs significantly increased in patients with pancreatic ductal adenocarcinoma 113. Upregulation of let-7 family miRNAs can inhibit K-Ras gene activity, thereby affecting cancer cell proliferation and migration. In addition, quercetin-induced miR-200b-3p converts symmetric cell division to asymmetric cell division by reversing the Notch/Numb ratio; inhibiting self-renewal; and activating the potential of cells to differentiate into adipocytes, osteocytes, and chondrocytes 115. Furthermore, quercetin induces miR-16 expression in human cancer. High miR-16 expression can inhibit claudin-2, a mediator of intestinal barrier leakage during intestinal inflammation 116. Among these quercetin-regulated microRNAs, let-7 family miRNA and hsa-miR-200b target TMPRSS2 and ACE-2, respectively 43, 45. Furthermore, has-miR-16 expression suppresses the formation of structural proteins by directly targeting nucleocapsids 56. By modulating miRNA expression, quercetin may prevent SARS-CoV-2 cell entry and replication. Overall, the daily dietary intake of quercetin can prevent SARS-CoV-2 infection and slow COVID-19 progression by modulating let-7 family miRNA, hsa-miR-200 family miRNA, and hsa-miR-16 expression (Figure 6).

**Epigallocatechin-3-gallate (EGCG)** is a flavanol consisting of a catechin conjugated with gallic acid. EGCG is abundant in green tea and cocoa-based products 117. In vivo and in vitro studies have reported the inhibitory effects of EGCG on various viruses, including SARS-CoV-2. Henss et al. reported that EGCG exhibit anti-SARS-CoV-2 activity by inhibiting the interaction between S proteins and ACE2 118. Furthermore, molecular docking revealed that EGCG has a high binding affinity with the SARS-CoV-2 S protein 119. EGCG effectively inhibited SARS-CoV-2 M^pro^ activity and HCoV-229E replication in Huh-7 cells at a dose of 2.5 μM 111. Henss et al. reported that EGCG effectively blocked the entry of SARS-CoV-2 into Vero E6 cells infected with the SARS-CoV strain Frankfurt-1, with an IC_50_ of 1.72 µg ml^-1^ (Table 3) 118. Overall, EGCG might be suitable for use as a lead structure to develop more effective anti-COVID-19 drugs. Following treatment with quercetin and EGCG, hsa-let-7 family miRNA expression was specifically induced in lung cancer 113. In another study, when EGCG was used in the IL-1β-stimulated human osteoarthritis chondrocyte cell line, it modulated the expression of various miRNAs, including let-7 family miRNAs, hsa-miR-125b-5p, and hsa-miR-497 120. Li et al. reported that EGCG mitigated Aβ-induced neurotoxicity by inducing miR-34a-5p and miR-125b-5p expression 121. In addition, EGCG acted as an anti-cervical cancer agent by suppressing cervical carcinoma cell growth by upregulating hsa-miR-125b and hsa-miR-203 expression, indicating that EGCG has therapeutic potential for use in the prevention of cervical cancer 122. Moreover. EGCG upregulated the expression of hsa-miRNA-15b in both murine and human T cells 123. Shin et al. reported that EGCG protected against the effects of dihydrotestosterone-induced apoptosis and reduced intracellular ROS levels by altering the miRNA expression profile of human dermal papilla cells 124, wherein the expression of hsa-miR-3613-3p was significantly upregulated following treatment with EGCG. hsa-miR-3613 was predicted to bind to the 3′-UTR region of the SARS-CoV-2 genome, suggesting that EGCG prevents viral replication by targeting the sequences of replication-related SARS-CoV-2 RNAs 67. hsa-let-7 family of miRNAs can suppress *TMPRSS2* expression by binding to the 3′-UTR region of *TMPRSS2*. In addition, hsa-miR-125b-5p can suppress *ACE2* expression by targeting 3′-UTR of *ACE2*, thus decreasing the likelihood of SARS-CoV-2 cell entry. In addition, hsa-miR-497-5p targets the RNA sequence of the SARS-CoV-2 S protein and may therefore inhibit the production of the S protein during the SARS-CoV-2 life cycle, resulting in the reduced yield and assembly of viral proteins. These findings suggest that EGEG interacts with SARS-CoV-2 and host cells by regulating let-7-family miRNAs, hsa-miR-15b, and hsa-miR-125b to inhibit viral entry into cells and regulating hsa-miR-497-5p expression, thus restricting production of the S protein (Figure 6).

**Resveratrol (3,40,5-trihydroxystilbene)** belongs to the stilbene class and thus exists in both cis and trans forms. It is abundant in grapes, grape juice, and red wine 125. Similar to EGCG, resveratrol exerts inhibitory effects on various viruses including SARS-CoV-2. At a dose of 66 µM, resveratrol exerted a dose-dependent antiviral effect on SARS-CoV-2 in Vero E6 cells and reduced virus particle production by 50% 126. In another study, resveratrol significantly suppressed SARS-CoV-2 replication in Vero E6 cells infected with SARS-CoV-2 (BetaCoV/Shenzhen/SZTH-003/2020 strain; Table 3), with a half-maximal effective concentration of 4.48 μM 127. Accumulating evidence indicates that resveratrol inhibits major pathways involved in the pathogenesis of SARS-CoV-2 by modulating the expression of *ACE2* and suppressing the release of proinflammatory cytokines and the production of ROS 128. In one study, resveratrol reduced the titer of SARS-CoV-2 with minimal cytotoxic effects 129. Therefore, resveratrol may be used alone or in combination with FDA-approved drugs to treat COVID-19 130. Resveratrol/CM-glucan formulations may be suitable for the simultaneous volatilization of aerosols in the treatment of patients with lower respiratory SARS-CoV-2 infections 131. Overall, resveratrol may reduce the spread of SARS-CoV-2 in the lower respiratory tract and inhibit viral replication in the early stages of infection 10. Fu et al. reported that resveratrol inhibits pancreatic cancer growth and metastasis by upregulating the expression of hsa-miR‑200 family members 132. Han et al. reported that resveratrol suppressed growth inhibition by inducing hsa-miR-622 expression in 16HBE-T cells 133. Furthermore, resveratrol exhibited neuroprotective properties in pneumococcal meningitis by modulating global miRNA expression, such as by upregulating the expression of hsa-miR-15b-5p, hsa-miR-25-3p, and hsa-miR-125b-5p. Among these resveratrol-induced miRNAs, hsa-miR-200 family miRNA and hsa-miR-125b expression can suppress *ACE2* expression. Furthermore, hsa-miR-15b and hsa-miR-622 bind to the SARS-CoV-2 S protein. Taken together, these findings suggest that resveratrol can regulate miRNA expression, thus preventing SARS-CoV-2 infection by inhibiting viral entry and viral replication (Figure 6).

**Curcumin** is mainly present in turmeric plants and curry powders and is not widespread in food 107. One clinical trial revealed that that treating COVID-19 with nanocurcumin significantly reduced the serum concentrations of IL-6 and IL-1β 134, 135. One in vitro study reported that curcumin (10 µg/mL) could inhibit 99% and 99.8% of the viral activity of the DG614 strain and Delta variant of SARS-CoV-2, respectively 136. In another study investigating the effect of turmeric root extract on SARS-CoV-2, curcumin achieved the complete neutralization of SARS-CoV-2 at a subtoxic concentration of 15.6 µg/mL (Table 3) 137. Goc et al. reported that at concentrations of 10 to 25 μg/mL, curcumin inhibited 20% to 30% of ACE2 activity in cell-based assays (Table 3) 138. Another study reported that curcumin exhibited a high binding affinity to the protease of SARS-CoV-2, indicating that curcumin may be suitable for use in the development of drugs that can prevent the host cell entry and replication of coronaviruses 139. Curcumin treatment can induce the expression of hsa-miR-9, hsa-miR-200 family miRNAs, hsa-miR-203, hsa-miR-16, and hsa-let-7a in cancer cells 140, 141. In an orthotopic xenograft model of human pancreatic cancer, curcumin treatment inhibited tumor growth by inducing hsa-let-7 expression 142. These results indicate that curcumin can protect against SARS-CoV-2 entry by inducing the expression of let-7 family miRNAs, which suppress *TMPRSS2* expression (Figure 6). In addition, curcumin treatment upregulated the expression of hsa-miR-199 and hsa-miR-200 in vivo, suggesting that curcumin treatment is beneficial for liver fibrosis 143. In leukemia cells, curcumin treatment significantly increased the expression of hsa-miR-16, which induced cell death by apoptosis and suppressed cell proliferation 144. Hsa-miR-16 upregulation was also observed in human breast cancer cells treated with curcumin 145. In another study, curcumin directly induced the expression of the tumor-suppressive microRNA hsa-miR-203 in bladder cancer by hypomethylating the hsa-miR-203 promoter 146. Curcumin suppressed the expression of genes encoding amyloid precursor proteins and amyloid-β and upregulated the expression of hsa-miR-15b-5p in swAPP695-HEK293 cells 147. Taken together, the curcumin-induced expression of hsa-miR-15b, hsa-miR-200 family miRNA, and hsa-miR-125b suppressed the expression of ACE2 and the S protein gene, indicating that curcumin can protect against SARS-CoV-2 infection (Figure 6).

The aforementioned natural compounds exhibit potential for use as anti-SARS-CoV-2 infection agents because they reduce infection-induced inflammation and ROS generation and prevent interactions between SARS-CoV-2 and host cells. Combined use with antiviral drugs can synergistically improve the efficiency of these agents in protecting against SARS-CoV-2 with minimal side effects. Using molecular docking approaches, some studies have demonstrated that flavonoids target ACE-2 and S proteins. Among these flavonoids, quercetin, EGCG, curcumin, and resveratrol exhibit high binding affinities to ACE-2 receptors and thus inhibit the entry of SARS-CoV-2 into host cells 148-152. In addition to directly binding to ACE-2/S proteins to prevent SARS-CoV-2 entry, miRNAs can protect against SARS-CoV-2 by downregulating the expression of *ACE2*, *TMPRSS2*, and the S protein gene. Herein, we summarize recent findings indicating that these natural compounds protect against SARS-CoV-2 entry host cells by modulating miRNA expression (Figure 6).

## Conclusions

Antiviral drugs and vaccines are key cost-effective tools for controlling the COVID-19 pandemic. However, the emergence of SARS-CoV-2 variants could threaten the global impact of mass vaccination and the effectiveness of antiviral drug development. Therefore, adjunctive therapy with nutritional polyphenolic compounds would be a beneficial alternative. As miRNAs are involved in SARS-CoV-2-induced immune evasion and cytokine storm, this contributes to the progression of COVID-19. Polyphenols are known to modulate miRNAs in various disease entities, including RNA virus infection. In this article, we preliminarily explore the prospective roles of miRNAs in the pathogenesis of SARS-CoV-2, including host cell miRNA-human gene interactions, human miRNA-SARS-CoV-2 transcript interactions, SARS-CoV-2 derived RNA transcripts act as decoy human miRNAs, and SARS-CoV-2 miRNA-human gene interactions. Polyphenolic compounds can prevent SARS-CoV-2 infection and inhibit SARS-CoV-2 replication by modulating host miRNAs. Suppressing infection and replication of SARS-Cov-2 can reduce the amount of cellular ceRNA, thereby maintaining the normal physiological function of the host miRNAs. Certain circulating miRNAs might be useful biomarkers of COVID-19 or be used in alternative approaches to COVID-19 treatment. In this paper, we describe four polyphenols, namely quercetin, EGCG, curcumin, and resveratrol, that are present in foods and may provide prophylactic or therapeutic benefits for SARS-CoV-2 infection by modulating host miRNAs. In the future, additional biological experiments and clinical trials must be conducted to further clarify these findings and to further elucidate the role of these miRNAs in the pathogenesis and treatment of COVID-19.

## Figures and Tables

**Table 1:** Human miRNAs directly bind to the RNA transcripts of SARS-CoV-2.

SARS-VoV2	miRNAs	Reference
S protein	hsa-miR-510-3p, hsa-miR-624-5p, hsa-miR-497-5p, hsa-miR-622, hsa-miR-761, hsa-miR-15b-5p, hsa-miR-196a-5p, hsa-miR-565, hsa-miR-151a-5p, hsa-miR-380-5p, hsa-miR-449a, hsa-miR-4464, hsa-miR1234-3p, hsa-miR-7107-5p, hsa-885-5p	53-55
Replication related RNA	hsa-miR-1307-3p	67
3' untranslated region	hsa-miR-3613-5p, hsa-miR-8066	79
NSPs/ORF1a/b	hsa-miR-203b-3p, hsa-miR-103a-1-5p, hsa-miR-6818-5p, hsa-miR-624-5p, hsa-miR-378c, hsa-miR-3202, hsa-miR-5591-5p, hsa-miR-8082, hsa-miR-939-5p, hsa-miR-549a-3p and hsa-miR-6515-5p	7, 58
ORF8	hsa-miR-12129, hsa-miR-2392, hsa-miR-23b-5p and hsa-miR-5047	7
Nucleocapsid	hsa-miR-21-3p, hsa-miR-195-5p, hsa-miR-16-5p, hsa-miR-3065-5p, hsa-miR-424-5p, hsa-miR-421, hsa-miR-6817-5p, hsa-miR-506-3p and hsa-miR-12119	7, 56

**Table 2:** Circulating miRNAs identified as biomarkers for diagnosis and prognosis of patients with COVID-19

	microRNA	Clinical samples	References
Diagnosis markers	hsa-miR-10b, hsa-miR-21, hsa-miR-155, hsa-miR-208a, hsa-miR-499, hsa-miR-29a-3p, hsa-miR-146a, hsa-miR-155-5p, hsa-miR-2392, hsa-miR-155, hsa-miR-19a-3p, hsa-miR-19b-3p, hsa-miR-92a-3p, hsa-miR-150-5p, hsa-miR-375, hsa-miR-122-5p, hsa-miR-494-3p, hsa-miR-3197, hsa-miR4690-5p, hsa-miR-1915-3p and hsa-miR-3652	COVID-19 v.s healthy control	69, 89, 91-93
	hsa-miR-106b-5p, hsa-miR-221-3p, hsa-miR25-3p, hsa-miR-30a-5p	COVID-19 v.s Community-acquired pneumonias	88
	hsa-miR-155, hsa-miR-208a, hsa-miR-499	COVID-19 v.s Influenza-ARDS	90
Prognosis markers	miR-nsp3-3p, hsa-miR-320a/b/c, hsa-miR-200c, hsa-miR-155	Mild/moderate patients v.s severe patients	80, 89, 96, 97
	hsa-miR-29a-3p, hsa-miR-146a-3p	Acute phase v.s post-acute pahase	91
	hsa-miR-146a-5p	Drug response	98
	hsa-miR-148a-3p, hsa-miR-451a, hsa-miR-486-5p, hsa-miR-2392	ICU v.s ward patients	92, 99
	hsa-miR-192-5p, hsa-miR323a-3p	ICU survivors v.s non-survivors	99
	hsa-miR-320b, hsa-miR483-5p	Survivors v.s non-survivors	100

**Table 3:** In vitro studies investigating the effects of polyphenols against SARS-CoV-2 infection

Polyphenols	Cell Model	SARS-CoV-2 strain	Concentration for used	References
Quercetin	Huh-7	Human coronavirus 229E	2.5uM~50uM	111
	Vero E6 cells	SARS-CoV-2 strain 026V-03883	Compound 1: 200uM~500uM; Compound 2d: 10uM~100uM	112
EGCG	Huh-7	Human coronavirus 229E	2.5uM~50M	111
	HEK293T-hACE2 and Vero E6 cells	SARS-CoV strain Frankfurt-1	1.25ug/ml ~25ug/ml	118
Resveratrol	Vero E6 cells, Calu-3 cells and primary human bronchial epithel (PBECs)	SARS-CoV-2 strain NL/2020	15uM~150uM	126
	Vero E6 cells	SARS-CoV-2 (BetaCoV/Shenzhen/SZTH-003/2020 strain)	1.56uM~200uM	127
Curcumin	hACE2/A549	eGFP-luciferase-SARS-CoV-2 pseudo-typed particles	2.5~100 ug/ml	138
	Vero E6 cells,	SARS-CoV-2 D614G strain and Delta variant	1.25~10 ug/ml	136
	Vero E6 cells and Calu-3 cells	SARS-CoV-2 isolated form hospital	1ug/ml~125ug/ml	137
